# Motivations and segmentation of instagram users as a social network site: Their sociodemographic profiles, satisfaction and loyalty

**DOI:** 10.1371/journal.pone.0354487

**Published:** 2026-07-30

**Authors:** Mauricio Carvache-Franco, Ana Beatriz Hernández-Lara, Orly Carvache-Franco, Wilmer Carvache-Franco

**Affiliations:** 1 Universidad Bolivariana del Ecuador, Campus Durán Km 5.5 Vía Durán Yaguachi, Durán, Ecuador; 2 Universidad ESAN, Graduate School of Business, Av. Alonso de Molina, Santiago de Surco, Lima, Peru; 3 Universitat Rovira i Virgili, Departament de Gestió d’Empreses, Avinguda Universitat, Reus, Spain; 4 Universidad Espíritu Santo, Km. 2.5 Vía a Samborondón, Samborondón, Ecuador; 5 Escuela Superior Politécnica del Litoral, ESPOL. Facultad de Ciencias Sociales y Humanísticas, Campus Gustavo Galindo Km Vía Perimetral, Guayaquil, Ecuador; UMass Amherst: University of Massachusetts Amherst, UNITED STATES OF AMERICA

## Abstract

Millions of Internet users interact daily with social network sites (SNS), and these figures are still expected to grow. This study aimed to identify the motivations and typology of different types of Instagram users, specifically pursuing these objectives: (1) to identify the motivations that lead individuals to use Instagram as a SNS; (2) to establish user segments based on these motivations; (3) to determine how these segments relate to user satisfaction and loyalty; and (4) to explore the relationship between the identified segments and key socio‑economic and demographic variables. The study was carried out through an online survey composed of 369 participants. For data analysis, factorial analysis, the K-means segmentation method, and the Chi-square test were used. Four motivational dimensions in the use of Instagram were identified, related to “Curiosity, Creativity, and Documentation,” “Self-expression,” “Socialization,” and “Recreation.” In addition, three distinct segments of Instagram users were identified based on their underlying motivations: “Enthusiasts”, “Spectators”, and “Passive Users”. Among them, the Enthusiasts emerged as the group displaying the highest levels of satisfaction and loyalty within the platform. The study further assessed the socio-economic and demographic profiles of each segment. This study offers original value by categorizing users based on their motivations, examining how these motivations relate to satisfaction and loyalty, and identifying the socio-economic and demographic profiles of the different user segments. The findings contribute to strategic frameworks for digital marketing and audience segmentation, providing organizations and businesses, particularly those that use Instagram as a communication, sales, or business channel, with actionable insights. Overall, this research establishes a solid foundation for future studies and practical applications in social media strategy, consumer behavior, and digital marketing.

## Introduction

Social Network Sites (SNS) are interactive communication technologies that facilitate creating and exchanging information, ideas, and other forms of expression through communities and virtual networks [1,2]. Approximately 3.6 billion internet users actively interact with SNS, and these figures are still expected to grow as the rise in mobile device creation makes social networks increasingly appealing [3].

SNS such as Instagram, Facebook, TikTok, and X have become deeply embedded in the daily lives of both young people [4,5] and adults around the world, evolving far beyond their original role as communication tools. Among them, Instagram stands out as a particularly influential platform, enabling users to share personal moments and transforming ordinary individuals into microcelebrities by turning content creation into a lifestyle [6,7]. The platform has also gained notable popularity among university students and women, with factors such as gender and privacy concerns shaping users’ preference for this social network [8].

This platform fosters social connection, creative self-expression, and the promotion of businesses and personal brands through its core activity of sharing images and videos. It also acts as a rich source of visual content, plays a role in shaping and disseminating cultural trends, and offers real-time entertainment through features like live streams. At the same time, concerns have been raised regarding its potential impact on mental well-being, particularly due to issues related to social comparison and self-image [9]. Ultimately, the significance of Instagram varies across individuals depending on their needs and expectations, yet it continues to hold a prominent place in contemporary digital sphere.

The use of Instagram offers a wide range of benefits across social, creative, and professional domains. Moreover, it can be observed that one of the platform’s predominant contemporary issues is engaging with brand-driven content and trends. Instagram hosts more than 25 million brand accounts, including 90% of the world’s top 100 brands [10]. Beyond its original function as a platform for sharing photos, it has evolved into a powerful national and international business space [11].

Several factors draw diverse users to this SNS. These include the appeal generated by influencers [12], the increasing presence of luxury brands that were initially reluctant to establish an online identity [13], and the growing number of entrepreneurial ventures operating on the platform. As a result, Instagram has solidified its role as a direct gateway between brands and consumers, becoming the fifth most-used social network worldwide in 2021 [14].

The growing influence of SNS on corporate marketing strategies has fostered a research line aimed at better understanding the potential of these platforms to enhance sales and business performance. This research has been interested on users’ motivations and the segmentation of social media audiences [15–18]. However, although some studies have examined user segmentation based on motivations, this body of research remains relatively scarce. A closer review shows a clear need for further contributions on the motivations driving Instagram use and, in particular, on the motivationally based user segments that can be identified on this platform. Additionally, there is a notable gap concerning how these segments relate to users’ socio-economic and demographic characteristics, as well as their implications for satisfaction and loyalty.

This study seeks to address these gaps by examining Instagram users’ motivations, identifying user segments derived from their motivational profiles, assessing how these segments differ in terms of user satisfaction and loyalty, and analyzing the socio‑demographic patterns associated with each group. Building on these aims, the present work deepens the understanding of the factors driving Instagram’s widespread adoption and the benefits users derive from engaging with the platform. Accordingly, the objectives of this article are: a) to identify the motivations of Instagram users; b) to establish a segmentation of users based on their motivational profiles; c) to determine the relationship between these segments and users’ satisfaction and loyalty; and d) to explore the association between the identified segments and key socio-economic and demographic variables.

By gaining a deeper understanding of what motivates different types of users to engage with Instagram in today’s society, we can better recognize the platform’s significance, how and why people use it, as well as its broader implications for communication, culture, and business in the digital era.

## Literature review

### Instagram as a SNS

Instagram was founded on October 6, 2010, by Kevin Systrom and Mike Krieger [19]. Initially conceived as an online social networking service designed primarily for mobile devices, it enables users to share photos and videos in an intuitive, visually oriented format [20]. In its early description, Instagram presented itself as “a fun and quirky way to share your life with friends through photos. Take a picture with your mobile phone and choose a filter to turn the image into a memory you can keep forever” [21].

In line with its original emphasis on visual storytelling, Instagram enables users to share photos and videos accompanied by hashtags (#), which facilitate content discovery and help organize posts around themes or trends. The platform also functions as a full social networking service, allowing users to create personal profiles and establish “follower” relationships with other users. These connections are inherently asymmetrical, as users are not required to reciprocate follow requests [22]. Today, sharing images through SNS like Instagram has become widespread and culturally embedded practice [22].

One distinguishing feature of Instagram, in contrast to other SNS such as Facebook, is that content creation is predominantly based on the sharing of visual material, requiring users to upload photographs or videos when publishing posts [23]. In addition, the platform incorporates built-in filtering tools that enable users to modify and aesthetically enhance their visual content prior to publication. Furthermore, Instagram users often maintain public accounts, enabling interaction mechanisms, such as following, viewing, liking, and commenting, between individuals who are not part of the same immediate social circles [23].

### Motivations for using instagram

As one of the world’s most widely used social platforms, Instagram shapes how people communicate, express themselves, and interact with brands and communities. Its broad user base and strong influence on cultural trends and digital identity prompt an essential question: *why do people use Instagram?*. Understanding these motivations is crucial given the platform’s integration into users’ daily routines, its strategic importance for brand building and audience engagement, and its impact on the performance of organizations operating within it. These factors highlight the need to explore the motives that drive Instagram use and the benefits and gratifications users derive from the platform [24].

Different studies have attempted to identify the motivations that drive the use of SNS. For example, [24] found that “Surveillance/Knowing about others”, “Documentation”, “Freshness”, and “Creativity” are the most relevant motivations explaining the use of Instagram, with “Surveillance/Knowing about others” being the most influential of the four. In a study by [25] they found that “Social interaction” is an important motivation and justified it with phrases like “To see what others share,” “To like my followers’ photos,” and “To follow my friends.” Furthermore, [25] found other motivations classified as “fun” through parameters like “To avoid loneliness,” “To relax,” and “To escape reality,” as well as the “creativity” motivation through elements like “To create art” and “To show my photographic skills.” Additionally, both studies, [24] and [25], found that the variable “documentation” constitutes an important motivation for using Instagram, broken down into elements such as “To remember special events,” “To commemorate an event,” and “To remember something important.”

[12] through a study with users between the ages of 20 and 39, found five social and psychological motivations in the use of SNS: “social interaction”, “archiving”, “self-expression”, “escapism”, and “sneaky peeks”. “Social interaction” entails connecting with others, staying informed about friends and family, and nurturing interpersonal relationships. “Archiving” involves documenting daily experiences, curating a personal digital space, and preserving photos and memories. “Self‑expression” reflects the desire to communicate one’s authentic identity, present oneself to others, and share personal content. “Escapism” denotes the use of the platform to momentarily detach from reality or alleviate loneliness. “Sneaky peeks” describes the inclination to observe aspects of others’ day‑to‑day lives. Collectively, these motivations promote more favorable attitudes toward Instagram and strengthen users’ intentions to continue using the platform [12].

Additionally, [26] found five motivations for using Instagram: “social interaction”, “creativity”, “self-promotion”, “fun”, and “documentation”. The study also found that, among these motivations, the most prominent were social interaction and fun derived from passive engagement, particularly through viewing others’ posts.

On the other hand, [27] found that the motivations for using Instagram include “liking”, “viewing posts”, “perceived social support”, and “the drive that leads to frequent posting”. These results not only explain users’ motivation but also the frequency of use of this social network, acting as predictors of users’ well-being, both positive and negative. In the same year, a study by [16] dedicated to art on Instagram identified five social and psychological motivations primarily affecting Instagram users, which coincide with those previously mentioned by [12]: “social interaction”, “archiving”, “self-expression”, “escapism”, and “sneaky peeks”. Similarly, the study by [28] found six motivations for using Instagram: “self-expression”, “recreation”, “socialization”, “recording”, “creativity”, and “curiosity”.

More recent research on Instagram‑use motivations has moved beyond general analyses, instead examining motivation patterns within specific user cohorts or contextual factors, such as age, cultural background, or particular features of the platform.

Among the studies that consider specific features of the platform, those analyzing what motivates the use of Instagram Stories stand out, which constitutes one of the most attractive aspects of Instagram. Stories use a format known as IGTV (Instagram TV), which are Reels or videos with a maximum duration of 15 minutes. The study by [29] indicated that people were curious about these IGTVs, with their use primarily motivated by “users’ leisure time”, “social capital”, and “the informational value of the IGTVs”.

Among the studies that examine specific user cohorts or contextual factors shaping Instagram use, such as users’ age, one noteworthy contribution focuses on baby boomers [18], which found that motivations for using Instagram among older adults include: “relationship surveillance”, “documentation”, “inspiration”, “self-promotion”, and “fun/companionship”. Their results emphasize how the use of SNS, and in particular Instagram, helps alleviate loneliness and a lack of social activity in this age group. Based on the Uses and Gratifications framework, [24] also examined the underlying motivations for Instagram use and identified multiple dimensions, such as “social monitoring”, “self-documentation”, “creative expression”, and “perceived social appeal”. Their results indicate that these motivations vary significantly according to contextual age, suggesting that user engagement with Instagram is not static but changes throughout the life span in response to evolving social and psychological demands.

Similarly, non-generalist research in this area emphasizes the importance of considering cultural factors when understanding the use of SNS. For example, a study by [30] on the assessment of Instagram use across different cultures indicates that individuals turn to social networks to satisfy unmet social and psychological needs, with their motivations for use being strongly influenced by cultural factors. This finding is grounded in the influence of culture on individuals’ self‑perception, their interpretation of their environment, and the way they understand the situations around them, as well as on their broader phenomenological experiences.

The literature reviewed indicates that research on Instagram-use motivations has evolved over time. Early studies adopted broad, general approaches, identifying core motivations such as social interaction and fun, alongside other less dominant yet still relevant drivers, including documentation or archiving and creativity. More recent work has shifted toward more targeted analyses, focusing on specific aspects of the platform or on contextual and contingent factors such as users’ age or cultural background. However, despite this progress, these studies remain limited. Further research is needed to identify, on the one hand, the most transversal and influential motivations for Instagram use and, on the other, the specific patterns that emerge when socio-economic and demographic variables are taken into account, such as gender or professional and employability characteristics.

### User segmentation of instagram

Market segmentation analysis examines a market structure as consumers perceive it [31]. At its most basic level, the term “market segmentation” refers to the subdivision of a market based on some common element, similarity, or affiliation [32]. Market segmentation aims to identify and delimit market segments or “sets of buyers” that become targets for a company’s marketing plans [33]. The starting point in segmentation is the correct definition of the market, which is crucial for determining its size, growth, and a company’s specific share in it, identifying relevant competitors, and formulating strategies to offer a differential advantage [34]. It is also related to understanding the reasons behind customer behavior before seeking market segments based on the analysis of their needs [34].

SNS, and particularly Instagram, are gaining importance as platforms for social interaction, communication, marketing, and business. Understanding how Instagram users could be segmented is crucial, as this will help visualize the business significance that this social network represents at the corporate level. For this reason, a growing number of companies across all sectors have already integrated, or plan to integrate, social media applications into their marketing strategies.

Academic literature has not remained on the sidelines in the study of this phenomenon, and numerous scholarly works have analyzed the segmentation of SNS users over the last decades. However, this research has relied predominantly on behavioral criteria, with most studies grouping users according to their frequency of use, the variety of activities performed, and/or their level of interaction and engagement, basing segmentation largely on behavioral parameters.

For example, early works as those by [35,36] and [37] classify users as occasional (named as “beginners”, “introvert”, “novel”, or “basic”), moderate (labelled as “versatile”, “social users” and “average”), or intensive (recognized as “expert-communicator”, “outstanding” and “expert”) based on how often they access social media and the extent to which they perform actions such as browsing, commenting, or posting content. Engagement-based distinctions, such as “sporadic”, “lurkers” (or passive users), “socializers” (mainly using SNS for social interaction), and “active contributors” (identifying people actively and frequently uploading content and writing contributions), are also common, as highlighted in [38] typology. Some studies refine behavioral analysis by examining interaction metrics, including likes, comments, and content evaluations, as in [39] segmentation of fan page users.

Beyond behavioral parameters, a smaller group of studies incorporates psychographic variables such as attitudes, involvement, or satisfaction in using SNS, as seen in [15], who identified five consumer segments for their responsiveness to marketing communications in SNS. Also, [28] conducted a segmentation analysis considering key context-specific usage motives of Instagram. They identified a first group, labeled “passionate”, characterized by the strongest motivational drive across all use motives. This was the only segment that clearly valued Instagram’s benefits for self-expression and recreation. The second group, termed “distant”, consisted of users who showed very low inclination to seek benefits related to self-expression, recreation, or creativity, and who held generally neutral views about the platform’s value for recording or socializing. The third group, called “spectator”, considered self-expression and recreation as largely irrelevant motives for them. This study also aimed at figuring out the main personality traits differences among Instagram users.

Finally, more recent research introduces demographic dimensions, notably gender, age, and generational cohorts, to identify differences in SNS adoption and acceptance. For example, [17] identified four groups using a priori segmentation based on gender and generation, considering four groups, two referred to baby boomers, men and women (born between 1947 and 1966) and two referred to the silent generation, men and women (born between 1927 and 1946), and analyzed the predictors of acceptance of social networking sites within these groups. In a similar vein, [40] found, based on technological preparedness profiles and generation, three segments with different determinants to explain the intention to use social media: “independent older adults,” “technologically apathetic older adults,” and “tech-enthusiast older adults.”

Overall, existing studies on segmentation have relied predominantly on behavioral indicators, while the incorporation of motivational and demographic factors remains comparatively limited. The incorporation of other criteria, like broader motivational dimensions, and socio-economic and demographic characteristics would complement the abundant segmentation work focused on frequency and patterns of use, offering a more comprehensive understanding of the heterogeneity of social media users.

### Sociodemographic aspects, satisfaction and loyalty of Instagram users

Contemporary social media research increasingly underscores the value of incorporating sociodemographic variables into SNS user segmentation to better capture differences in usage patterns and interaction motivations.

Recent studies indicate that Instagram usage profiles are closely associated with factors such as age, gender, and income, enabling the identification of segments with distinct behaviors and needs within platform [41]. Complementing this perspective, emerging research has begun to examine motivational profiles, for example, among university students, showing that segments such as passive users versus reciprocal communicators differ not only in their platform engagement but also in their interests, for example to discuss public, social and political issues, highlighting the interplay between motivational and demographic characteristics [42]. Similarly, [43] demonstrated that followers of Instagram influencers can be grouped into differentiated segments based on consumption patterns, including the newly identified “Mirror Tourist,” a profile that blends interests in fashion and tourism and is shaped by underlying demographic attributes.

Together, these findings suggest that segmentation strategies should integrate both sociodemographic and motivational data to provide more accurate and actionable insights, particularly in business and marketing contexts. Ultimately, segmentation models that incorporate sociodemographic dimensions not only enhance academic understanding of Instagram use but also equip social and commercial organizations with more precise tools for designing communication strategies tailored to specific audiences.

In the same vein, studies on motivations and user segmentation in SNS should be complemented by analyses of their effects on user satisfaction and loyalty, as these outcomes help explain how different user profiles reinforce continued platform use [17,36,40]. For example, [15] in a study examining 1,025 social media users across four regions, identified five consumer segments based on their responsiveness to marketing communications, incorporating psychographic variables such as attitude, involvement, satisfaction, and intention to continue using SNS. Their findings illustrate how specific user segments are linked to satisfaction and loyalty metrics, an insight particularly relevant for companies and brands operating on these platforms.

In conclusion, although motivations play a central role in shaping user behavior on social networking sites (SNS), segmentation research has largely overlooked motivation-based criteria. Only a limited number of recent studies, most notably [28], explicitly incorporate motivational factors, highlighting the need for additional research that can corroborate or further refine the motivational segments identified so far in order to assess the most transversal and influential motivations among Instagram users. Moreover, existing research rarely examines how motivational patterns differ across socio-demographic groups, nor does it sufficiently explore how these user segments relate to key outcome variables such as satisfaction and loyalty. These gaps limit our ability to generate actionable managerial insights, as companies lack the knowledge needed to understand the specific traits and motivations of user segments as well as their likelihood of continued Instagram use.

## Methodology

This research is part of a project approved by the Ethics Committee of the University ESPOL and its Ethics Committee, under the code CERT-PI-CEIE-003–2023. As part of the ethical procedures, informed consent was included in the questionnaire used for data collection, which was previously accepted by the participants in written form.

To achieve the objectives of this study, a quantitative research approach was used by applying a questionnaire, which was designed to encompass several sections related to the objectives. First, the socioeconomic and demographic characteristics of the respondents were collected through closed-ended questions adapted from the study by [44]. Secondly, a 23-item Likert scale of motivations (where 1 was “not important” and 5 was “very important”) was used, adapted from the study by [28]. The reliability of the motivation scale was evaluated using Cronbach’s alpha coefficient, resulting in a value of 0.96, indicating high internal consistency among the items that made up the scale.

Thirdly, questions about general satisfaction and intentions to recommend the use of Instagram were included. Satisfaction was evaluated using a 5-point Likert scale, ranging from 1 (not satisfied) to 5 (very satisfied). Similarly, loyalty, determined by intentions to recommend the use of Instagram, was measured using a 5-point Likert scale with different labels (strongly disagree = 1, to strongly agree = 5). The scales assessing satisfaction and loyalty were adapted from the research by [45].

The sample was obtained through online surveys designed using Google Forms. Participants were over 18 years old and used Instagram as a SNS. The sample was collected online using WhatsApp from April 1 to May 30, 2024. The questionnaire was initially distributed to the researchers’ close contacts via WhatsApp. Participants were then invited to share the questionnaire with their own social and professional networks. This approach allowed for the gradual expansion of the sample, facilitating access to a broader and more diverse group of respondents. As a result, data were obtained from individuals from different backgrounds, increasing the sample’s variability while maintaining the feasibility of data collection in an online environment. The participants in the sample reside in Ecuador. The sample was collected using a non-probabilistic convenience sampling approach. However, during the selection process, efforts were made to maintain a balanced distribution of participants across key sociodemographic variables, age, gender, educational level, and occupation, in order to approximate the profile of the target population and mitigate potential biases associated with online data collection. This study used a sample size of 369 participants, with a sampling error of 5%, an acceptable value for this type of study. The population variability was set at 50% (p = q = 0.5) for the sample size. Additionally, a 5% margin of error and a 95% confidence level were considered. The data were organized, tabulated, and analyzed in SPSS IBM version 26, comprising three stages of analysis.

Initially, the underlying constructs of the variables were identified through factor analysis. The application of the Varimax rotation resulted in a simple factor structure, characterized by the concentration of high loadings on a single factor. Kaiser’s criterion helped determine the number of factors to retain, specifically those with eigenvalues greater than 1. In particular, the Kaiser-Meyer-Olkin (KMO) index and Bartlett’s sphericity test indicated the suitability for performing this analysis.

In the second stage, a K-means clustering method was used to identify the different segments according to Instagram usage as a social network. The Kruskal-Wallis H test was applied to find the differences between the means of the three segments. This test indicated that the three segments differed but did not specify where these differences lie. For this, the Mann-Whitney U test was used to find the differences between the means of two segments, according to different combinations (significant difference between segment 1 and segment 2; significant difference between segment 1 and segment 3; significant difference between segment 2 and segment 3).

The third stage used the Chi-square test to analyze the existence of significant relationships (p < 0.05) between the segments and the satisfaction variables and intentions to recommend. Additionally, relationships between the segments and the socio-demographic variables were analyzed.

## Results

### Sample profile

According to the sample, the percentage of women was slightly higher than that of men. A high percentage (61.4%) of respondents were between 18 and 23 years old. A large percentage were single (81.7%). The majority of the sample had a high educational level (university) (73.5%). Most participants were students (63.1%) with incomes of less than 500 dollars (67.9%) and used Instagram for more than 60 minutes a day (33.5%). See Table 1.

**Table 1:** Socio-demographic variables.

Variable	Categories	Percentage
Gender	Male	42.3
	Female	57.7
Age	Between 18 and 23 years old	61.4
	Between 24 and 29 years old	19.4
	Between 30 and 35 years old	9.3
	Between 36 and 41 years old	5.1
	More than 41 years old	4.8
Marital Status	Single	81.7
	Married	14.1
	Other	4.2
Level of education	Primary	0.3
	Secondary	16.1
	University	73.5
	Postgraduate/ PhD	10.1
Occupation	Student	63.1
	Business owner	4.8
	Private employee	24.8
	Public employee	7.3
Monthly income	Less than 500 USD	67.9
	From 500 to 1.000 USD	25.4
	From 1.000 to 1.500 USD	4.8
	More than 1.500 USD	2
Time used per day on Instagram	Less than 10 minutes	11.3
	10–30 minutes	28.5
	31–60 minutes	26.8
	More than 60 minutes	33.5

### Motivations of Instagram users

A factor analysis was performed to identify fewer factors that could explain the items related to Instagram usage motivations. The factor loadings ranged from 0.501 to 0.810, with all values exceeding 0.5. Cronbach’s alpha values ranged from 0.926 to 0.889, all high and close to 1. This indicated a high level of reliability among the items within each factor. The KMO index reached a value of 0.939. Additionally, Bartlett’s test of sphericity was significant (sig = 0.00). These values indicated the appropriateness of the factor analysis.

In the results, no substantial differences were observed between primary and secondary loadings. All items exhibited a dominant factor with loadings above 0.50, along with adequate communality values. Items belonging to the same construct showed high and homogeneous loadings without dispersion across multiple factors, providing evidence of satisfactory convergent and discriminant validity of the factorial model. See Table 2. The Table with the full factor loadings is presented in Appendix A in S1 Appendix.

**Table 2:** Motivations of Instagram users.

Variables	Curiosity, Creativity, Documentation	Self-Expression	Socialization	Recreation
To explore a variety of stylish photos	0.810			
To showcase my photographic skills	0.728			
To record daily events through photos	0.719			
To create art	0.700			
To search for photos related to my interests	0.655			
To find people with whom I share common interests	0.623			
To remember and commemorate special events	0.606			
To take stylish photos and store them online	0.520			
To track my journey (e.g., travel) through a photo map	0.501			
To become popular		0.818		
To be noticed by others		0.776		
To share personal information with others		0.708		
To express my true self (who I am)		0.660		
To create personal blogs		0.628		
To provide “visual status updates” to my friends		0.607		
To keep in touch with friends who are far away			0.878	
To communicate with friends and family			0.833	
To receive updates about close friends and family			0.732	
To maintain good relationships with others (for networking)			0.619	
To escape from reality				0.794
To avoid loneliness				0.792
To forget about problems				0.785
To relax				0.655
Eigenvalues	11.877	1.827	1.546	1.129
Cronbach’s Alpha	0.926	0.914	0.881	0.889
Explained Variance (%)	51.637	7.943	6.721	4.911
Cumulative Variance (%)	51.637	59.580	66.301	71.211

According to Table 2, the first dimension was named “Curiosity, Creativity, and Documentation” because it is related to the use of Instagram for recording and remembering events, creating art, showcasing photographic skills, and exploring through photography. This factor explained 51.64% of the variance. The second dimension was named “Self-Expression” because it is related to becoming popular, being noticed, and expressing one’s identity. This factor explained 7.94% of the variance. The third dimension was “Socialization” because it relates to interaction with friends, family, and networking. This dimension accounted for 6.72% of the variance. The fourth dimension was named “Recreation” because it is related to escaping from reality and relaxing. This dimension captured 4.91% of the variance.

### Segmentation by Instagram user motivations

A K-means segmentation has been used to analyze the different segments based on Instagram usage. See Table 3. The three-cluster solution was examined using a dendrogram, which supported the presence of three distinct groupings. See Appendix B in S2 Appendix.

**Table 3:** Segmentation by Instagram user motivations.

Variable	Enthusiasts	Spectators	Passives	Kruskal-Wallis H	P-value	Mann-Whitney U
To be noticed by others	3.8	2.0	1.5	167.453	0.000	All < 0.05
To become popular	4.0	1.9	1.4	180.007	0.000	All < 0.05
To share personal information with others	3.9	2.4	1.7	158.378	0.000	All < 0.05
To express my true self (who I am)	4.2	2.4	1.6	186.477	0.000	All < 0.05
To provide “visual status updates” to my friends	4.3	3.0	1.7	191.410	0.000	All < 0.05
To take stylish photos and store them online	4.4	3.4	2.1	148.304	0.000	All < 0.05
To forget about problems	3.9	2.4	1.8	130.143	0.000	All < 0.05
To avoid loneliness	3.7	2.1	1.4	151.130	0.000	All < 0.05
To escape from reality	3.8	2.2	1.5	154.951	0.000	All < 0.05
To relax	4.4	3.5	2.5	117.380	0.000	All < 0.05
To receive updates about close friends and family	4.2	3.6	2.5	116.391	0.000	All < 0.05
To keep in touch with friends who are far away	4.3	3.8	2.5	119.445	0.000	All < 0.05
To communicate with friends and family	4.2	3.6	2.3	123.458	0.000	All < 0.05
To maintain good relationships with others (for networking)	4.3	3.1	1.9	161.506	0.000	All < 0.05
To document my tracks (e.g., travel) through a photomap	4.2	3.1	1.6	178.205	0.000	All < 0.05
To make personal blogs	4.0	2.3	1.3	204.161	0.000	All < 0.05
To record daily events through photos	4.1	3.0	1.4	211.817	0.000	All < 0.05
To remember and commemorate special events	4.3	3.4	1.9	179.077	0.000	All < 0.05
To create art	4.1	2.5	1.5	183.852	0.000	All < 0.05
To showcase my photographic skills	4.0	2.8	1.3	192.294	0.000	All < 0.05
To find people with common interests	4.2	2.8	1.9	169.357	0.000	All < 0.05
To explore a variety of stylish photos	4.2	3.1	1.8	172.483	0.000	All < 0.05
To search for photos related to my interests	4.4	3.5	2.3	144.788	0.000	All < 0.05

According to Table 3, the first segment was labeled “Enthusiasts” because it had high scores across all motivations, making it a group excited to use Instagram for all the reasons included in this study. This segment particularly values Instagram’s benefits of self-expression and recreation, seeking a high and proactive presence on the platform. The second group, “Observers,” showed high motivation levels related to socialization, curiosity, creativity, and documentation. However, motivations related to self-expression and recreation were less important, differentiating them from the Enthusiast segment. This group is more motivated to follow what others are doing than to share images, activities, or personal memories. The third segment was called “Passive” because it scored low on all motivations, indicating that they use Instagram but not much or have limited interest.

On the other hand, the Kruskal-Wallis test showed significant results below 0.05 for all items, meaning there was a significant difference between the means of the three segments, indicating that the segments are distinct. However, this test does not indicate where those significant differences lie. The Mann-Whitney U test was used for that purpose, and it showed significant differences below 0.05 in all the combinations of two segments (Segment 1 and Segment 2; Segment 2 and Segment 3; Segment 1 and Segment 3). This indicated a significant difference in all two-segment combinations for each item.

### Relationship between Instagram user segmentation and satisfaction and loyalty

The Chi-square test was used to analyze whether significant relationships existed between the segments, the satisfaction variables, and the intentions to recommend. See Table 4.

**Table 4:** Relationship of the segments with satisfaction and loyalty.

Variable	Enthusiasts	Spectators	Passives	Chi-square	P-value
Satisfaction with the use of Instagram as a social network	4.1622	3.9767	3.487	40.061	0.000
Intention to recommend the use of Instagram as a social network	4.4324	3.8915	3.3043	71.801	0.000

According to the results in Table 4, the “Enthusiasts” segment showed the highest level of satisfaction and loyalty based on their intention to recommend Instagram. This result is not surprising given the diversity of motivations linked to this segment, which should be the focus of marketing policies for organizations on this platform. This is due to this group’s proactivity in recommending the social network and the viral effects that might be associated with these recommendations.

### Relationship of the segments with socio-economic and demographic variables

The chi-square test was used to test whether segment membership is statistically associated with socio-economic and demographic attributes, revealing systematic differences in how these characteristics are distributed across segments. See Table 5.

**Table 5:** Relationship of the segments with socio-economic and demographic variables.

Variables		Enthusiasts	Spectators	Passives	Chi-square	P-value
Age	Between 18 and 23 years	49.5%	70.5%	62.6%	19.625	0.012
	Between 24 and 29 years	29.7%	13.2%	16.5%		
	Between 30 and 35 years	13.5%	5.4%	9.6%		
	Between 36 and 41 years	4.5%	4.7%	6.1%		
	Over 41 years	2.7%	6.2%	5.2%		
Marital Status	Single	75.7%	87.6%	80.9%	13.400	0.009
	Married	22.5%	8.5%	12.2%		
	Other	1.8%	3.9%	7.0%		
Occupation	Student	49.5%	69.0%	69.6%	22.841	0.001
	Business owner	2.7%	3.9%	7.8%		
	Private employee	37.8%	18.6%	19.1%		
	Public employee	9.9%	8.5%	3.5%		
Monthly income	Under 500 USD	56.8%	71.3%	74.8%	18.727	0.005
	500 to 1.000 USD	34.2%	21.7%	20.9%		
	1.000 to 1.500 USD	9.0%	3.1%	2.6%		
	More than 1.500 USD		3.9%	1.7%		
Time of Instagram Usage	Less than 10 minutes	3.6%	11.6%	18.3%	52.576	0.000
	10 - 30 minutes	13.5%	33.3%	37.4%		
	31–60 minutes	26.1%	26.4%	27.8%		
	More than 60 minutes	56.8%	28.7%	16.5%		

According to Table 5, Chi-square tests showed significant associations between segment membership and age (χ² = 19.63, *p* = .012); marital status (χ² = 13.40, *p* = .009); occupation (χ² = 22.84, *p* = .001); monthly income (χ² = 18.73, *p* = .005); and time of Instagram use (χ² = 52.58, *p* < .001), indicating non‑random distributions of socio‑demographic characteristics and usage intensity across segments.

“Spectators” skew younger (18–23: 70.5%) compared with “Enthusiasts” (49.5%) and “Passives” (62.6%). “Enthusiasts” show relatively higher representation in the 24–29 (29.7% vs. 13.2–16.5%) and 30–35 brackets (13.5% vs. 5.4–9.6%), suggesting a comparatively older profile. “Spectators” are most likely to be single (87.6%), whereas “Enthusiasts” include a larger share of married users (22.5%). “Spectators” and “Passives” are predominantly students (69.0% and 69.6%, respectively), while “Enthusiasts” include more private (37.8%) and public employees (9.9%), consistent with a more established occupational profile. Lower income levels (<USD 500) are most prevalent among “Passives” (74.8%) and “Spectators” (71.3%), and least prevalent among “Enthusiasts” (56.8%). The “Enthusiasts” segment had the highest percentage of people spending more than 60 minutes on Instagram daily, making this segment the most engaged social media platform.

## Discussion

This paper explores what motivates Instagram use and the benefits it provides to its users through motivation analysis and segmentation. It also relates the segments to socio-economic and demographic variables, as well as user satisfaction and loyalty.

Regarding the first objective, identifying motivations for using Instagram as a social network, four main motivations were identified, ranked by the variance they captured: “Curiosity, creativity, and documentation,” related to the use of Instagram for recording and remembering events, creating art, and exploring through photography; “Self-expression,” related to issues of identity and empowerment of the “self”; “Socialization,” linked to motivations for interpersonal relationships; and “Recreation,” associated with motivations for fun, escape, and relaxation.

These results align with previous researches analyzing general motivations for using Instagram and other social networks. For example, “Documentation” (also referred to as “recording” or “archiving”) was identified as a key motivation for using Instagram in studies by [24,25,27,28], and [18]. What is interesting and original about our study is that motivations related to curiosity, creativity, and documentation are grouped into a single motivating factor, as they are conveyed through a common medium: photography. Other studies identify these as separate and independent motivations, such as [24,25,27] who distinguish between “documentation” and “creativity,” or [28], who separate “recording,” “creativity,” and “curiosity” as distinct motivations.

On the other hand, the “Self-expression” motivation was also highlighted in the studies by [12,16], and [28] in [27] and [18] under the label “self-promotion,” and by [26], recognizing it as “perceived social support.”

The “Socialization” motivation was emphasized in the studies by [24] and [18] under the label of surveillance (of relationships) and knowledge of others; [12,25,27], and [16] referred to it as social interaction; [28] directly identified it as socialization; and [29] used the social capital factor to identify this motivation for using Instagram.

Finally, the “Recreation” motivation was also identified in various studies that labeled it as freshness [24], fun [27; 25], escapism [12,16], recreation [28], or camaraderie [18].

Another contribution of our results on motivation analysis is that, while other works highlight motivations related to social interaction [27; 24,25; 18] and fun [27], in our sample, the motivations that capture the most variance are those related to curiosity, creativity, and documentation, which connect a range of uses related to Instagram and based on the creation, sharing, and exploration of photographs, as a distinctive feature of this social network.

Regarding the second objective of the study, analyzing the segmentation of Instagram’s use as a social network, our results revealed three user segments labeled “Enthusiasts,” “Spectators,” and “Passive.” Most previous segmentation studies apply a frequency-based approach, distinguishing users who mainly create content from those who only consume/view it or engage based on interaction on the platform through likes, comments, or user-generated content. This approach is evident in research such as [37], which identifies basic, average, outstanding, and expert users; [38], which classifies users as sporadic, lurkers, or advanced; or [39], which distinguishes users by engagement, identifying groups like Apathetic Fans, Staunch Fans, Ordinary Fans, and Lazy Fans.

This study complements a smaller number of works considering users’ motivations when segmenting them. One such study is [35], which identifies three types of users linked to specific motivations: those interested in social interaction, information seeking, and sharing photos/videos. Other recent studies enrich segmentation by not linking each segment to a single motivation but rather a set of them with varying weights, identifying more multifaceted segments. This is the case with [28], whose results align closely with ours. In comparison with [28], our study contributes additional value by both corroborating and refining their motivational segmentation of Instagram users. While we replicate the general three-segment structure they identified, parallels to their “passionate,” “spectator,” and “distant” profiles, our results show more nuanced distinctions within these groups. In particular, we find a different internal hierarchy of motivations in the segments, for example, whereas the “distant” group prioritizes socialization and documentation, our “Passive” users emphasize socialization and recreation. This differentiation illustrates that motivational profiles are not fixed but can vary across contexts, confirming the robustness of the overall segmentation while demonstrating that the relative weight of motivations within each segment may shift.

The third objective, exploring the relationship between segments and user satisfaction and loyalty on Instagram, represents a significant contribution to our work. It goes beyond previous studies by linking the identified segments with behavioral variables related to platform use and the possible virtualization of their content, analyzing how these segments relate to user satisfaction and their willingness to recommend the social network. Our results confirm that the most proactive users on the social network, and those who generate the most content, are the most satisfied and loyal; their loyalty is greater than that of users who act as mere followers or display a more passive attitude.

The final objective of this study, examining how the identified segments relate to key socio-economic and demographic variables, aligns with previous research that has primarily considered gender and age/generation when segmenting Instagram users or analyzing their motivations [17,40,46,47]. Our work extends these contributions by incorporating additional variables linked to users’ social, educational/professional, and economic profiles, enabling a more refined characterization of the platform’s most proactive user groups. The results show that the “Enthusiast” segment, which spends the most time on the platform, tends to be slightly older than the average user and includes a higher proportion of married individuals, private-sector employees, and users with comparatively higher incomes.

## Conclusion

This work contributes to a better understanding of what motivates the use of Instagram among different types of users in contemporary society to appreciate the importance of this platform and the purposes it serves. What are the potential effects on marketing and business among these types of users?

Various implications arise from the results obtained. At the theoretical level, this work contributes to the state of the art on motivations for the use of Social Networking Sites (SNS), and Instagram in particular. Our results highlight the significant relevance of Instagram as a platform that fosters users’ creativity, exploration, and curiosity, primarily through the central role of photographs. In this sense, photographs become a key connecting element that shapes how users engage with the platform and how they mentally distinguish it from other social networks. Our findings also confirm the existence of different user types on this network, with different socio-economic and demographic profile, emphasizing the importance of identity construction and recreation as the predominant motivations among the most satisfied and influential users. These individuals tend to be the most proactive, showing a greater likelihood of creating and sharing content. In contrast, other users act more as followers and exhibit lower levels of satisfaction and loyalty, being motivated mainly by the opportunities for socialization, recreation, or documentation that the platform offers.

This study also provides meaningful practical implications for researchers, brand managers, and digital communication professionals who use Instagram as a strategic platform. The findings are valuable for all organizations with a presence on Instagram that aim to leverage the platform more effectively and increase the return on their digital efforts. In particular, companies and institutions will be especially interested in attracting the attention of the users we identify as “Enthusiasts,” who are the most proactive segment. Through their active role as content creators, these users show the highest levels of satisfaction and loyalty to the platform and have strong potential to amplify content virally through their recommendations. The identification of this enthusiastic segment, characterized by high interaction levels and motivated by self-expression and leisure-related benefits, highlights a clear preference for maintaining an active and participatory presence on the platform. For this group, communication strategies should emphasize participatory formats such as user-generated content initiatives, interactive polls, live streaming, and influencer collaborations. Given their high engagement intensity, “Enthusiasts” may act as organic disseminators of brand messages, making it essential to encourage two-way communication and loyalty-building efforts that support long-term engagement.

The “Spectator” segment, which shows strong motivation linked to socialization, curiosity, creativity, and content documentation, represents an audience with substantial activation potential. For these users, strategies should prioritize informative content, coherent visual storytelling, and moderate calls to action that facilitate a gradual transition toward more active participation. In this context, performance indicators such as viewing duration, reach, and save rates may offer more accurate insights than traditional direct-interaction metrics when evaluating the effectiveness of initiatives aimed at this group.

Finally, “Passive” users, who demonstrate low motivational intensity, require strategies focused primarily on increasing reach and brand awareness rather than immediate conversion. For this segment, content should be concise, visually appealing, and frequently repeated, supported by paid campaigns, short-format posts, and emotionally engaging messages that build brand familiarity without demanding high cognitive effort. This group is particularly relevant for long-term visibility and brand positioning objectives.

From an applied perspective, these findings suggest that organizations should adopt differentiated segmentation strategies rather than uniform approaches that assume homogeneous user behavior on Instagram. Furthermore, integrating sociodemographic and behavioral variables can improve segmentation accuracy and support more efficient allocation of communication resources.

However, the findings of this research are subject to certain limitations, primarily related to the sample size and the number of motivations considered. The application of other methodologies, not based on information collection through questionnaires but through exploring the social network itself, which allows for capturing motivations and user profiles via Big Data tools, would provide more complete information and avoid the risks inherent in surveys. Looking to the future, we propose complementing and triangulating the results of this study by collecting information with these Big Data tools, expanding the sample, and applying more sophisticated analyses to consider greater variability in Instagram use among its users. Including a more significant number of socio-economic and demographic variables, cultural variables, and other impact variables beyond satisfaction and loyalty would be useful to more precisely capture the return that different types of organizations and companies achieve through their presence on Instagram.

## Supporting information

S1 Appendix Appendix A. Table of the Motivations of Instagram Users (Factor Analysis, full factor loadings).
(DOCX)

S2 Appendix Appendix B. Dendrogram to determine the number of clusters. “Motivations Instagram Database.xlsx”.
(DOCX)

S1 Data Motivations Instagram Database. (XLSX)

## References

[1] Ahn J, Son H, Chung AD. Understanding public engagement on twitter using topic modeling: The 2019 Ridgecrest earthquake case. International Journal of Information Management Data Insights. 2021;1(2):100033. doi: 10.1016/j.jjimei.2021.100033

[2] Kietzmann JH, Hermkens K, McCarthy IP, Silvestre BS. Social media? Get serious! Understanding the functional building blocks of social media. Business Horizons. 2011;54(3):241–51. doi: 10.1016/j.bushor.2011.01.005

[3] Kar AK. What Affects Usage Satisfaction in Mobile Payments? Modelling User Generated Content to Develop the “Digital Service Usage Satisfaction Model”. Inf Syst Front. 2021;23(5):1341–61. doi: 10.1007/s10796-020-10045-0 32837261 PMC7368597

[4] Lakhiwal A, Kar AK. Insights from Twitter Analytics: Modeling Social Media Personality Dimensions and Impact of Breakthrough Events. Lecture Notes in Computer Science. Springer International Publishing. 2016. p. 533–44. doi: 10.1007/978-3-319-45234-0_47

[5] Van Dijck J, Poell T. Understanding social media logic. Media and Communication. 2013;1(1):2–14.

[6] Morshed TB, Hernández-Lara AB. Women travelers and social media: Charting the path to economic and entrepreneurial opportunities. Journal of Destination Marketing & Management. 2024;34:100952. doi: 10.1016/j.jdmm.2024.100952

[7] Morshed TB, Hernández-Lara AB. Exploring the role of female travel influencers: A network and thematic analysis. Social Sciences & Humanities Open. 2025;11:101583. doi: 10.1016/j.ssaho.2025.101583

[8] Shane-Simpson C, Manago A, Gaggi N, Gillespie-Lynch K. Why do college students prefer Facebook, Twitter, or Instagram? Site affordances, tensions between privacy and self-expression, and implications for social capital. Computers in Human Behavior. 2018;86:276–88. doi: 10.1016/j.chb.2018.04.041

[9] Faelens L, Hoorelbeke K, Cambier R, van Put J, Van de Putte E, De Raedt R, et al. The relationship between Instagram use and indicators of mental health: A systematic review. Computers in Human Behavior Reports. 2021;4:100121. doi: 10.1016/j.chbr.2021.100121

[10] Smith K. 50 incredible Instagram statistics. https://www.brandwatch.com/blog/instagram-stats/ 2019.

[11] Rodríguez JG, Barth M, Fischer D. Evolution of entrepreneurs’ expectations using Instagram as a business practice: A transformative learning perspective in the case of sustainable fashion entrepreneurs in Mexico. World Development Sustainability. 2022;1.

[12] Lee E, Lee JA, Moon JH, Sung Y. Pictures speak louder than words: motivations for using Instagram. Cyberpsychology, Behavior and Social Networking. 2015;18(9):552–6. doi: 10.1089/cyber.2015.0157 26348817

[13] Oliveira M, Fernandes T. Luxury brands and social media: drivers and outcomes of consumer engagement on Instagram. Journal of Strategic Marketing. 2020;30(4):389–407. doi: 10.1080/0965254x.2020.1777459

[14] Castillo‐Abdul B, Pérez‐Escoda A, Núñez‐Barriopedro E. Promoting social media engagement via branded content communication: A fashion brands study on Instagram. Media and Communication. 2022;10(1):185–97. doi: 10.17645/mac.v10i1.4728

[15] Andrews L, Bianchi C, Wiese M, Cuneo A, Fazal E. Hasan S. Segmenting Brands’ Social Network Site (Sns) Consumers: A Four-Country Study. Journal of International Consumer Marketing. 2018;31(1):22–38. doi: 10.1080/08961530.2018.1475273

[16] Kang X, Chen W, Kang J. Art in the Age of Social Media: Interaction Behavior Analysis of Instagram Art Accounts. Informatics. 2019;6(4):52. doi: 10.3390/informatics6040052

[17] Ramírez-Correa PE, Rondán-Cataluña FJ, Arenas-Gaitán J, Grandón EE, Alfaro-Pérez JL, Ramírez-Santana M. Segmentation of Older Adults in the Acceptance of Social Networking Sites Using Machine Learning. Front Psychol. 2021;12:705715. doi: 10.3389/fpsyg.2021.705715 34456818 PMC8385199

[18] Sheldon P, Antony MG, and Ware LJ. Baby Boomers’ use of Facebook and Instagram: uses and gratifications theory and contextual age indicators. Heliyon, 2021; 7(4). doi: 10.1016/j.heliyon.2021.e06670 PMC8049997 33889780

[19] Ha A. An experiment: Instagram marketing techniques and their effectiveness. California: California Polytechnic State University. 2015. http://digitalcommons.calpoly.edu/comssp/185

[20] Frommer D. Here’s how to use Instagram. Business Insider. 2010;:1–23.

[21] Dornan R. Reflecting the museum: How Instagram brings back seeing. Museum-iD magazine. 2016;(18).

[22] Zappavigna M. Social media photography: construing subjectivity in Instagram images. Visual Communication. 2016;15(3):271–92. doi: 10.1177/1470357216643220

[23] Chen H. College-Aged Young Consumers’ Perceptions of Social Media Marketing: The Story of Instagram. Journal of Current Issues & Research in Advertising. 2017;39(1):22–36. doi: 10.1080/10641734.2017.1372321

[24] Sheldon P, Bryant K. Instagram: Motives for its use and relationship to narcissism and contextual age. Computers in Human Behavior. 2016;58:89–97. doi: 10.1016/j.chb.2015.12.059

[25] Sheldon P, Rauschnabel PA, Antony MG, Car S. A cross-cultural comparison of Croatian and American social network sites: Exploring cultural differences in motives for Instagram use. Computers in Human Behavior. 2017;75:643–51. doi: 10.1016/j.chb.2017.06.009

[26] Huang YT, Su SF. Motives for Instagram use and topics of interest among young adults. Future Internet. 2018;10(8):77. doi: 10.3390/fi10080077

[27] Wong D, Amon KL, Keep M. Desire to belong affects Instagram behavior and perceived social support. Cyberpsychology, Behavior and Social Networking. 2019;22(7):465–71. doi: 10.1089/cyber.2018.0533 31295026

[28] Huang YT, Su SF. Motives for Instagram use and topics of interest among young adults. Future Internet. 2018;10(8):77. doi: 10.3390/fi10080077

[29] Kocak E, Nasir VA, Turker HB. What drives Instagram usage? User motives and personality traits. Online Information Review. 2020;44(3):625–43. doi: 10.1108/OIR-08-2019-0260

[30] Ko HC, Yu DH. Understanding continuance intention to view Instagram stories: A perspective of uses and gratifications theory. In: Proceedings of the 2nd International Conference on Control and Computer Vision, 2019. 127–32. 10.1145/3341016.3341039

[31] Schaffer DR, Debb SM. Assessing Instagram use across cultures: A confirmatory factor analysis. Cyberpsychology, Behavior and Social Networking. 2020;23(2):100–6. doi: 10.1089/cyber.2019.0247 31913711

[32] Johnson RM. Market Segmentation: A Strategic Management Tool. Journal of Marketing Research. 1971;8(1):13. doi: 10.2307/3149720

[33] Thomas JW. Market segmentation. Quarterly Review of Marketing. 1980;6(1):25–8.

[34] Tynan AC, Drayton J. Market segmentation. Journal of Marketing Management. 1987;2(3):301–35. doi: 10.1080/0267257x.1987.9964020

[35] McDonald M. Market segmentation. The marketing century. 2012. p. 27–50. doi: 10.1002/9781119208501.ch2

[36] Constantinides E, Zinck Stagno MC. Potential of the social media as instruments of higher education marketing: a segmentation study. Journal of Marketing for Higher Education. 2011;21(1):7–24. doi: 10.1080/08841241.2011.573593

[37] Alarcón-del-Amo M-C, Lorenzo-Romero C, Gómez-Borja M-Á. Classifying and profiling Social Networking Site users: a latent segmentation approach. Cyberpsychol Behav Soc Netw. 2011;14(9):547–53. doi: 10.1089/cyber.2010.0346 21288133

[38] Lorenzo-Romero C, Alarcón-del-Amo MC. Segmentation of users of social networking websites. Social Behavior and Personality: an international journal. 2012;40(3):401–14. doi: 10.2224/sbp.2012.40.3.401

[39] Brandtzaeg PB. Social Networking Sites: Their Users and Social Implications - A Longitudinal Study. J Comput-Mediat Comm. 2012;17(4):467–88. doi: 10.1111/j.1083-6101.2012.01580.x

[40] Khobzi H, Teimourpour B. LCP segmentation: A framework for evaluation of user engagement in online social networks. Computers in Human Behavior. 2015;50:101–7. doi: 10.1016/j.chb.2015.03.080

[41] Ramírez-Correa PE, Arenas-Gaitán J, Rondán-Cataluña FJ, Grandon EE, Ramírez-Santana M. Adoption of social networking sites among older adults: The role of the technology readiness and the generation to identifying segments. PLoS One. 2023;18(4):e0284585. doi: 10.1371/journal.pone.0284585 37071653 PMC10112803

[42] Pessoa K, Alves Costa CL, Coelho AC, Bastos A, Rodrigues I. Use of Instagram as a Resource for the Adoption of Behaviors Related to Health and Well-Being of Young College Students: Associations between Use Profile and Sociodemographic Variables—A Cross-Sectional Study. Societies. 2023;13(2):45. doi: 10.3390/soc13020045

[43] Choi M, Hong Y, Kwon H. Identifying Instagram user profiles: Who uses Instagram to get current public issues?. Atlantic Journal of Communication. 2024;33(2):295–310. doi: 10.1080/15456870.2024.2431288

[44] Sánchez-Amboage E, Crespo-Pereira V, Membiela-Pollán M, Jesús Faustino JP. Tourism marketing in the metaverse: A systematic literature review, building blocks, and future research directions. PLoS One. 2024;19(5):e0300599. doi: 10.1371/journal.pone.0300599 38728243 PMC11086888

[45] Lee TH, Jan F-H, Tseng CH, Lin YF. Segmentation by recreation experience in island-based tourism: a case study of Taiwan’s Liuqiu Island. Journal of Sustainable Tourism. 2017;26(3):362–78. doi: 10.1080/09669582.2017.1354865

[46] Kim K-H, Park D-B. Relationships Among Perceived Value, Satisfaction, and Loyalty: Community-Based Ecotourism in Korea. Journal of Travel & Tourism Marketing. 2016;34(2):171–91. doi: 10.1080/10548408.2016.1156609

[47] Schehl B, Leukel J, Sugumaran V. Understanding differentiated internet use in older adults: A study of informational, social, and instrumental online activities. Computers in Human Behavior. 2019;97:222–30. doi: 10.1016/j.chb.2019.03.031

