# Supplementary material for: Motivations and segmentation of instagram users as a social network site: Their sociodemographic profiles, satisfaction and loyalty
Source: PLoS One. 2026 Jul 30;21(7):e0354487. doi: 10.1371/journal.pone.0354487 (PMC13423056; doi:10.1371/journal.pone.0354487)
Supplement: S1 Appendix — Table of the Motivations of Instagram Users (Factor Analysis, full factor loadings). (DOCX) [file pone.0354487.s001.docx]

**Appendix A**

Table of the Motivations of Instagram Users (Factor Analysis, full factor loadings)

| **Variables** | **Curiosity, Creativity, Documentation** | **Self-Expression** | **Socialization** | **Recreation** |
| --- | --- | --- | --- | --- |
| To explore a variety of stylish photos | **0.810** | 0.135 | 0.199 | 0.283 |
| To showcase my photographic skills | **0.728** | 0.369 | 0.151 | 0.202 |
| To record daily events through photos | **0.719** | 0.406 | 0.256 | 0.094 |
| To create art | **0.700** | 0.370 | 0.141 | 0.171 |
| To search for photos related to my interests | **0.655** | 0.023 | 0.288 | 0.388 |
| To find people with whom I share common interests | **0.623** | 0.278 | 0.220 | 0.307 |
| To remember and commemorate special events | **0.606** | 0.341 | 0.410 | 0.034 |
| To take stylish photos and store them online | **0.520** | 0.424 | 0.317 | 0.082 |
| To track my journey (e.g., travel) through a photo map | **0.501** | 0.452 | 0.426 | 0.026 |
| To become popular | 0.276 | **0.818** | 0.060 | 0.269 |
| To be noticed by others | 0.240 | **0.776** | 0.110 | 0.285 |
| To share personal information with others | 0.156 | **0.708** | 0.245 | 0.279 |
| To express my true self (who I am) | 0.325 | **0.660** | 0.169 | 0.334 |
| To create personal blogs | 0.471 | **0.628** | 0.184 | 0.208 |
| To provide "visual status updates" to my friends | 0.387 | **0.607** | 0.360 | 0.160 |
| To keep in touch with friends who are far away | 0.225 | 0.092 | **0.878** | 0.151 |
| To communicate with friends and family | 0.191 | 0.158 | **0.833** | 0.194 |
| To receive updates about close friends and family | 0.247 | 0.159 | **0.732** | 0.256 |
| To maintain good relationships with others (for networking) | 0.314 | 0.372 | **0.619** | 0.175 |
| To escape from reality | 0.225 | 0.329 | 0.151 | **0.794** |
| To avoid loneliness | 0.168 | 0.400 | 0.135 | **0.792** |
| To forget about problems | 0.126 | 0.311 | 0.178 | **0.785** |
| To relax | 0.327 | 0.062 | 0.301 | **0.655** |
| Eigenvalues | 11.877 | 1.827 | 1.546 | 1.129 |
| Cronbach’s Alpha | 0.926 | 0.914 | 0.881 | 0.889 |
| Explained Variance (%) | 51.637 | 7.943 | 6.721 | 4.911 |
| Cumulative Variance (%) | 51.637 | 59.580 | 66.301 | 71.211 |
