# Supplementary figures and images for: Motivations and segmentation of instagram users as a social network site: Their sociodemographic profiles, satisfaction and loyalty
Source: PLoS One. 2026 Jul 30;21(7):e0354487. doi: 10.1371/journal.pone.0354487 (PMC13423056; doi:10.1371/journal.pone.0354487)

**Appendix B**

Dendrogram to determine the number of clusters


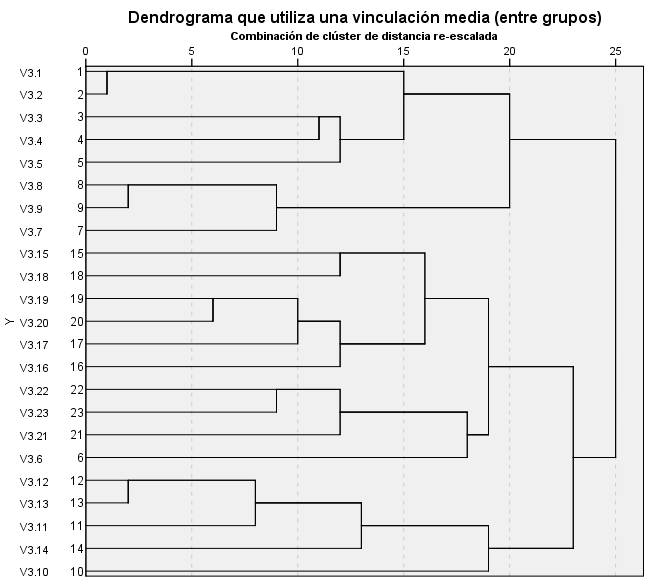

Supplement: S2 Appendix — Dendrogram to determine the number of clusters. “Motivations Instagram Database.xlsx”. (DOCX) [file pone.0354487.s002.docx]
